# Developing a Brief Tele-Psychotherapy Model for COVID-19 Patients and Their Family Members

**DOI:** 10.3389/fpsyg.2021.784685

**Published:** 2021-12-02

**Authors:** Bruno Biagianti, Silvana Zito, Chiara Fornoni, Valeria Ginex, Marcella Bellani, Cinzia Bressi, Paolo Brambilla

**Affiliations:** ^1^Department of Pathophysiology and Transplantation, University of Milan, Milan, Italy; ^2^Department of Neurosciences and Mental Health, Fondazione IRCCS Ca’ Granda, Ospedale Maggiore Policlinico, Milan, Italy; ^3^Department of Neurosciences, Section of Psychiatry, Biomedicine and Movement Sciences, University of Verona, Verona, Italy

**Keywords:** COVID-19, tele-psychiatry, treatment development, digital mental health intervention, psychotherapy

## Abstract

**Objective:** The COVID-19 pandemic is negatively impacting the mental health of COVID-19 patients and family members. Given the restrictions limiting in person contact to reduce the spread of the virus, a digital approach is needed to tackle the psychological aftermath of the pandemic. We present the development of a brief remote psychotherapy program for COVID-19 patients and/or their relatives.

**Methods:** We first reviewed the literature on psychotherapeutic interventions for COVID-19 related symptoms. Based on this evidence, we leveraged ongoing clinical experiences with COVID-19 survivors and family members to design an intervention model that could be disseminated and integrated into the workflow of the mental health system.

**Results:** This 8-session model –inspired by constructivist and hermeneutic-phenomenological therapies– serves COVID-19 patients during hospitalization, remission and recovery. This model can also be delivered to people dealing with the COVID-19 hospitalization/discharge of a family member, or the loss of a family member due to COVID-19.

**Conclusion:** We described a remote psychotherapeutic approach to tackle the COVID-19 pandemic psychological aftermath. To date, the approach seems feasible and highly customizable to patients’ needs. Studies are underway to test its preliminary efficacy. Once proven efficacious, this treatment model could provide a blueprint for future tele-psychology wide-scale interventions.

## Introduction

At the beginning of June 2021, the COVID-19 pandemic had affected 172,433,303 people worldwide, with 3,706,788 who died, and 155,086,546 who recovered. In Italy, out of a total of 60,379,981 inhabitants, 3,886,867 were infected and recovered from COVID-19^1^. On June 22nd, in the region of Lombardy, the total number of COVID-19 cases was 840,772, of which: 793,368 recovered, 33,757 died, while 13,647 were still positive cases^2^. These numbers not only describe the enormous impact of this phenomenon, but also shed light on its ongoing challenges.

Current predictive models show that during the (Dorman-Ilan et al., 2020) COVID-19 pandemic, the number of people with impaired mental health exceeded the number of people affected by the infection itself (Cannito et al., 2020; Delmastro and Zamariola, 2020; Fusar-Poli et al., 2020; Esposito et al., 2021). Those at greatest risk for mental distress are certainly COVID-19 patients and their family members (Landi et al., 2020; Tanoue et al., 2020). The psychological aftermath of this pandemic has been of unprecedented magnitude: COVID-19 survivors have risked their lives, have lost acquaintances or family members, and have spent long periods of isolation and social distancing, without being able to enjoy the physical presence of their significant others. Even during the recovery phase, the clinical management of COVID-19 sequelae and premorbid conditions has required the implementation of rehabilitation programs that have often neglected the psychological distress (Cereda et al., 2021; Negrini et al., 2021). Finally, despite the vaccination campaign, vulnerable individuals will likely have to live in conditions of partial or full restriction of their daily activities for a long time to come. On the other hand, family members and caregivers of COVID-19 survivors have experienced psychological distress during the hospitalization phase of their relatives, unable to offer personal assistance and receive adequate psychological support (Ying et al., 2020).

While many studies are available on the psychological impact the pandemic has had on the general population, few studies have considered survivors of COVID-19 (Cénat et al., 2021). The incidence and prevalence of psychological distress in COVID-19 patients varies by country and by study methodology (Cai et al., 2020), in a survey questionnaire study, report a psychological distress prevalence of 54.8% on a sample of 106 Chinese COVID-19 patients (mean age 45.7 ± 14) in early convalescence. Of these, 31, 22.2, and 38.1% met the criteria for excessive stress, anxiety, and depression, respectively. Authors highlight the difference with previous studies worldwide, where the incidence of mental disorders after major disasters ranged from 10 to 20%. Halpin et al. (2021) conducted a cross sectional study in the United Kingdom on 100 COVID-19 patients (68 ward, 32 Intensive Care Unit, mean age 64.5). PTSD symptoms were found in 23.5 ward patients and 46.9 ICU patients. Self-reported worsening of anxiety and depression was reported from 16.2 ward patients and 37.5 ICU. Finally, Imran et al. (2021) conducted a multicentric cross-sectional study in Dubai, with 103 and 85 COVID-19 patients evaluated at 4 and 8 weeks after discharge, respectively. They indicated a prevalence of anxiety symptoms (21.4 and 9.5%), depression (12.7 and 7.1%), and PTSD (8.7 and 4.7%). Unfortunately, no studies published to date indicate what is the prevalence and incidence of psychological stress in the relatives of COVID/19 survivors. In sum, the incidence and prevalence of psychological distress in COVID-19 survivors and their families have reached rates that go beyond the current possibilities of any mental health system to offer in-person services (Johnson et al., 2020) with the result that most users fail to receive the necessary support. For this reason, the public health systems of several countries have implemented *ad hoc* services for psychological support (Dong et al., 2020; Rosenberg et al., 2020). Since psychological suffering significantly reduces the quality of life (Corrigan and Buican, 1995; Choo et al., 2019) there exists an urgent need to intervene on the psychological suffering of COVID-19 survivors and their family members. Treatments that can be offered safely and on a large scale during the acute, weaning and recovery phases of the illness, without further burdening an already strained health system, are desperately needed (Fattori et al., 2021; Radfar et al., 2021).

Governments, policymakers, and stakeholders have called for action in this direction. Many countries have expanded their laws and regulations to allow for more widespread adoption of telemedicine systems by adding remote psychological therapies to their healthcare reimbursement lists. In Italy, on March 24th 2021 an open call for the adoption of telemedicine technologies and monitoring systems was jointly launched by the Ministry for Technological Innovation and Digitization, the Ministry of Health, the National Institute of Health and the World Health Organization^3^.

The purpose of this article is to describe our attempt to respond to this call to action. We first reviewed the scientific literature on psychotherapeutic interventions that were designed to treat symptoms among COVID-19 patients, family-members, and caregivers. Based on this evidence, we leveraged the ongoing clinical experiences with COVID-19 survivors and family members to design a clinical intervention model that could be remotely administered and could easily integrate with the reimbursement system of the regional health service. The result of this process was the development of a brief remote psychotherapy program to be delivered via telemedicine.

## State of the Art: Experimental Trials

The online search strategy was conducted through two of the major public scientific databases – PubMed and Scopus – and ended on May 30th, 2021. The following search terms were entered: (“COVID-19” OR “SARS-CoV-2” OR “coronavirus”) AND (“psychotherapy” OR “online psychological treatment” OR “online therapy”). No date limit was set and only contributions written in English were included. Gray literature was not searched for. Both single-arm and randomized controlled trials, as well as qualitative studies, were considered for eligibility. Abstracts, reviews, meta-analyses, opinion papers, research protocols, qualitative studies, and papers without outcome data were excluded.

Seven studies met our criteria and presented findings from psychological interventions delivered to COVID-19 patients (see Table 1). Unfortunately, no studies are available on interventions for family members.

**TABLE 1:** Psychological interventions delivered to COVID-19 patients.

Article	Study design	Sample (*n*)	Age (mean ± SD)	Treatment period	Psychological intervention	Severity of the disease	Pre-post quantitative measurements	Main results
Dincer and Inangil, 2021	Randomized controlled trial	35 Experimental 37 Control	Experimental: 33.37 ± 9.58 Controls: 33.54 ± 9.83	Single session, 7 subgroups of 5 patient participants	EFT	Exposed to COVID-19	Subjective units of Distress Scale State-Trait Anxiety Inventory Tx-1, Burnout scale	The intervention group experienced significant reductions in stress, anxiety, and burnout compared to the control group.
Ferrario et al., 2021	Case-control study	181	75.27 ± 12.45	Not reported	CBT	Post-Acute	//	Of 86 patients in the COVID-19 ward, 75.59% underwent psychological CBT treatment, 11.6% were supported remotely, only 7% showed good adaptation. While for the general number of this sample (181), 35.91% required structured psychological treatment.
Ping et al., 2020	Qualitative study	25	//	1 month	UBPI	Exposed to COVID	//	Nurses who used UBPI techniques for 1 month provided positive qualitative results
Shaygan et al., 2021	Pilot cluster randomized parallel-controlled trial	Experimental: 26 Controls = 22	36.77 ± 11.81	2 weeks, 14 daily modules	CBT	Mild-to-Moderate COVID-19 = 19 Severe COVID-19 = 7	Connor-Davidson Resilience Scale Perceived Stress Scale	The results suggest that compared to controls, the intervention group had significantly greater improvements in resilience and perceived stress scores after 2 weeks.
Sotoudeh et al., 2020	Randomized clinical trial	30	Experimental: 41.92 ± 12.2 Control: 44.7 ± 14.2	Not reported	Not reported	Acute	Depression, Anxiety and Stress Scale 21 Symptom Checklist (SCL-25), WHO-QOL-BREF	Significant differences were found between the experimental and control groups in terms of quality of life, depression, anxiety, stress, and mental health.
Wahlund et al., 2021	Randomized controlled trial	Intervention (*n* = 335) Waiting list (*n* = 335)	45 (13)	3 weeks	CBT	Daily worry about COVID-19	Generalized Anxiety Disorder Assessment (GAD-7) Work and Social Adjustment Scale Insomnia Severity Index Montgomery–Åsberg Depression Rating Scale Self Assessment	Treatment yielded significant outcomes by reducing concern about COVID-19 for the intervention group compared to waiting list, likewise on all secondary measures: mood, daily functioning, insomnia and intolerance to uncertainty.
Wei et al., 2020	Prospective, randomized, controlled, 2-week study	Intervention group (*n* = 13) Control group (*n* = 13)	Intervention group: 40.8 ± 13.5 Control group: 48.5 ± 9.5	2 weeks	Self-help intervention (desensitization techniques)	Medium COVID-19 = 5 Severe COVID-19 = 7	Hamilton Depression Rating Scale Hamilton anxiety rating Scale	COVID-19 patients in the intervention group showed significantly reduced levels of depression and anxiety symptoms after 2 weeks, compared with those in the control group.

*Abbreviations: CBT, Cognitive behavioral therapy; EFT, Emotional freedom techniques; SD, Standard deviation; and UBPI, Ultra brief psychological intervention.*

Three studies investigated the effects of self-guided, online programs designed to offer remote psychological assistance to COVID-19 patients (Wei et al., 2020; Shaygan et al., 2021; Wahlund et al., 2021). Wahlund et al. delivered a brief digital self-guided psychological intervention based on CBT principles to COVID-19 patients. The 3-week intervention was delivered through an encrypted study website. Results from this study showed significant reductions in COVID-19-related worries in patients compared to healthy controls, as well as in secondary outcomes (Wahlund et al., 2021). Wei and colleagues structured their intervention on audio-recordings that covered four main aspects: breathing relaxation training, awareness (body scanning), “refuge” ability, and the butterfly hug method (Wei et al., 2020). COVID-19 patients and healthy controls could access this self-help intervention for up to 2 weeks. Significant reductions in depression and anxiety were found in patients compared to controls. Shaygan et al. (2021) designed a 2-week online intervention centered around psychoeducational cognitive–behavioral techniques, stress management techniques, mindfulness-based stress reduction, and positive psychotherapy. After 2 weeks, COVID-19 patients showed greater resilience and less perceived stress compared to healthy controls. Sotoudeh et al. (2020) implemented a brief crisis intervention package that was adapted from brief CBT protocols and scientific publications in the realm of emergency psychiatry. The package was delivered at the psychological center of a public hospital where COVID-19 patients were hospitalized. The authors found a statistically significant reduction in anxiety, depression and perceived stress, as well as an improvement in quality of life and overall mental health among COVID-19 patients compared to healthy controls.

Ping et al. (2020) studied the effects of an Ultra Brief Psychological Intervention (UBPI) model that was conceptualized in 2018 for the Malaysian context. The UBPI model includes intervention techniques borrowed from different psychotherapeutic approaches, is based on indications from the Psychological First Aid, and was adapted to the unique needs emerging from the COVID-19 emergency. Ping and colleagues remotely delivered USPI over video on medical and nursing staff and on the general population. Findings from the study showed efficacy and acceptability, despite raising some ethical questions about the risks associated with the confidentiality of information.

Dincer and Inangil (2021) selected a sample of non-clinical subjects and conducted a singlesession, online, group-based intervention centered on the principles of emotionally focused therapy, which is recognized as a treatment for physical stress and emotional distress. Findings showed a significant reduction in perceived stress, anxiety, and burnout among COVID-19 patients compared to controls.

Ferrario et al. (2021) implemented a psychological intervention based on principles of CBT in a large sample of patients with COVID-19, who were assisted by a trained psychologist either remotely or in-person – directly from the COVID-19 ward where they were hospitalized. Results were reported somewhat inconsistently: of the 86 patients in the COVID-19 ward, 75.59% underwent psychological CBT treatment, 11.6% were supported remotely, only 7% showed good adaptation. As for the whole sample, authors reported that around 36% required structured psychological treatment, without reporting efficacy data.

## Construction of an Intervention Model

### Theoretical Framework

Our intervention model builds upon data currently existing in the literature, and harmonizes techniques and strategies deriving from several psychotherapeutic orientations (mainly of the third generation) into a brief psychotherapy program. The approaches that were integrated into the model are listed in Table 2. The combination of different approaches was necessary to overcome the limits imposed by the remote delivery (such as the reconfiguration of the setting) and the distance (ideal proxemic space). As a matter of fact, tele-therapy acutely limits the operationalization of therapeutic techniques based on in-person interaction, the sharing of the here and now, non-verbal and body language, with important implications on the development of transference and countertransference. Similarly, if on one hand tele-therapy allows patients and providers to bypass the risk (and/or fear) of exposure, on the other it makes it difficult to understand and deepen some inter-relational aspects. For example, our model does not consider the fluctuations of the relationship between the therapist and the patient along the way, such as the theme of building trust. Therefore, we considered it necessary to integrate various therapeutic techniques and strategies, in order to mitigate both the limitations presented by the screen and the difficulties in building a remote “connection.”

**TABLE 2:** Psychotherapeutic approaches taken into consideration when designing our theoretical framework.

Theoretical approach	Definition	Strategies and techniques used to design our model	References
Cognitive behavioral therapy (CBT)	CBT focuses on the assessment of change in behaviors, emotions, and cognitions	ABC technique; Socratic Colloquium; Gradual exposures to feared situations; Relaxation techniques associated with the imagination.	Andersson and Carlbring, 2017
Dialectical behavior therapy (DBT)	DBT aims to manage emotions and behaviors through “a balance and synthesis of both acceptance and change.” It uses the principles of (CBT) combined with awareness, acceptance, and dialectics.	Mindfulness, Tolerance to suffering, Regulation of emotions.	Eist, 2015; Huang et al., 2020
Compassion focused therapy (CFT)	CFT is an “integrated and multimodal approach that draws from evolutionary, social, developmental and Buddhist psychology, and neuroscience”	Evolutionary functional analysis of emotions: consult the safety/health function, tone of voice, look/smile Practice of perspective taking, recognition of challenges, letter of self-gratitude, compassionate images for oneself and others.	Gilbert, 2009
Acceptance and commitment therapy (ACT)	ACT aims to improve psychological flexibility, which refers to a person’s ability to connect with the present moment more fully as a conscious human being and to engage in value-based action	increase psychological flexibility through six interrelated fundamental processes: acceptance, defusion, contact with the present moment, self as context, values and committed action	Fiorillo et al., 2017; Apolinário-Hagen et al., 2020
Schema therapy (ST)	ST is an integrative treatment approach that combines cognitive, behavioral, experiential, and psychoanalytic therapy techniques.	“Bridge” between coping modalities that reflect emotional regulation strategies (overcompensation, avoidance, or surrender), current problems and personal history	Zens, 2019
Online eye movement desensitization and reworking (EMDR)	EMDR is developed to reduce processing intrusive traumatic memories	Float back of stressful events linked to Covid-19, Desensitization, psychoeducation and stabilization. “Safe place,” body scan and guided relaxation	Wilson et al., 2018; Tarquinio et al., 2020
Neuropsychological Cognitive Psychotherapy (PCN)	PCN integrates the most recent neuropsychological and biological knowledge are integrated with those deriving from the cognitive tradition, within a phenomenological- hermeneutic framework.	Imaginative Variation, Experience Refiguration, Experiential and Applied Learning	Liccione, 2013

Our model was developed along two main theoretical trajectories: constructivist therapy (Neimeyer and Mahoney, 1995; Neimeyer, 2001) and hermeneutic-phenomenological therapy (Chessick, 1993). Briefly, the constructivist model considers emotions in an evolutionary sense as an instrument of personal knowledge and growth. Constructive is also the connection between emotions and motivational systems (attachment system, fear of separation, and sadness-despair from loss). Given the short duration of the intervention, we considered it appropriate to exclude the constructivist part of attributing meaning to one’s emotional reaction in relation to one’s early life experiences. The hermeneutic-phenomenological approach assumes that, starting from the patient’s narration, the main issues to be addressed emerge spontaneously, along with any discrepancies between the patient’s lived experience and the factual nature of what they describe. According to this approach, the therapist highlights early on the patient’s ways of suffering and coping, in hopes to identify together with the patient which ones are already familiar and which ones are novel. In the context of the patient’s textual refiguration, by sharing clinical objectives the therapist makes room for interpretative cooperation: a commonality of intents that leads both, therapist and patient, to accept the fatigue of the clinical work (Ricœur, 1990). During all phases of the clinical work, suffering is contextualized both in the light of the recent traumatic experience (bereavement, hospitalization in intensive care, fear for one’s life or that of a relative), and in the light of historical ways of suffering, so that the patient is able to recognize the meaning of the symptoms experienced.

This model is designed to be disseminated on a large scale: in line with the reimbursement system of the regional health service of Lombardy, 8 remote, 50-min, individual psychological sessions are offered weekly using secure video conferencing software. The severity of the clinical conditions of COVID-19 patients has largely influenced the sequencing of the intervention both for patients themselves and for their family members. We considered it appropriate to circumscribe the exploration of the different psychological targets within each session, given the unpredictable nature of the course of illness, and the possible onset of events that radically change the psychological state of patients and family members. What follows is a very schematic summary that refers to the “ideal” situation, where clinical conditions (of the patient, or of the relative of the patient) evolve linearly toward recovery.

### Treatment Development

This model is designed to be applied to COVID-19 patients during hospitalization, after discharge, during the remission and recovery phases, as well as to COVID-19 survivors. Similarly, this model is intended to be delivered to people who are dealing with the hospitalization for/discharge after COVID-19 of a family member, or have lost a family member due to COVID-19. Based on our experience working with COVID-19 patients and family members, we have identified six clinical macro-areas that closely match those recently reported in the literature:

1) Traumatic and post-traumatic symptoms, mainly for those who have experienced hospitalization or are relatives of hospitalized patients (Cai et al., 2020; Kaseda and Levine, 2020). Emotional experiences that are often endorse include: worry and anxiety with catastrophic thoughts; moments of despondency and sadness (up to despair) with ideas of loss; anger, especially if the patient is young (anger from loss and anger from injustice); regret (for not having lived to the fullest the last time patient and family member saw each other); sense of guilt (for having transmitted the virus, for having contracted it in a less serious way). Guilt seems particularly relevant for family members, and often surfaces after moments of partial well-being during which they manage to distract themselves (as if to say it is not right to feel good while my family member is in the ICU);
2) For relatives of COVID-19 patients, suffering that originates from bereavement (Landa-Ramírez et al., 2020). Issues related to frozen grief: several patients also lost a parent to COVID-19, but when their significant other is hospitalized, it becomes more evident that they had frozen that grief, partially due to the absence of bereavement ceremonies;
3) Adaptation and functional reorientation due to somatic complications or sequelae (Ran et al., 2020). Many people affected by the COVID-19 experience directly or indirectly (as non-infected family members) report a subjective representation of reality characterized by recurrent, intrusive, and unwanted thoughts associated with the risk of/vulnerability to infection. Psychosomatic symptoms and self-perceived general health issues (gastrointestinal disturbances, fatigue, chest pains, palpitations, headaches, and difficulty falling asleep) are common and responsible for an increase in anxiety and an urge to use excessive and harmful protective measures even in safe places. Especially for COVID-19 survivors, the fear of the risk of returning to the experience of acute illness or contagion leads the subject to a constant body monitoring (control of blood pressure and saturation), to social and work avoidance, to the search for medical-clinical reassurance (emergency room visit, web searches) which reveal a strong desire to be assisted;
4) Identity reorientation following the traumatic experience (Halldorsdottir et al., 2021). In the younger age group, the theme of identity in development clearly emerges. A traumatic stress such as the COVID19 experience seems to undermine the construction and integration of aspects of the personality. Certainly, the short duration of intervention makes the psychological treatment of this macro-area and possible trajectories of identify development rather complicated;
5) Psychiatric symptoms triggered and/or exacerbated by the COVID-19 experience (Castellini et al., 2020; Belz et al., 2021). From a clinical point of view, the COVID-19 experience often exacerbates pre-existing symptoms. In other cases where subjects do not have a psychiatric history, pathological personological traits can emerge and require a range of flexible therapeutic strategies. In some cases, more serious symptoms such as depersonalization and derealization are the result of exacerbation of obsessive-compulsive personality traits that emerge because mechanisms of psychological resilience no longer are efficient.

### Treatment Manualization

Eight remote, 50-min, individual psychological sessions are offered weekly using secure video conferencing software. Please see Table 3 for a summary of the protocol.

**TABLE 3:** Goals, content, strategies, and techniques used in each session of the psychotherapeutic program.

Session number	Session goals	Session content	Strategies and techniques
1	Form the basis for therapeutic alliance validate the patient’s suffering offer closeness and support	Introductions exploration of the patient’s current experience space identification of the areas of suffering brief recapitulation of the patient’s psychological functioning pre-COVID	Collection of information regarding health conditions, clinical severity, length, and type of hospitalization float back of stressful events related to COVID-19 (situations, places, people, images) identification of variables that regulate symptomatology and functional mechanisms that underlie the lack of wellbeing
2	Learn how to regulate disruptive emotions reach the level of resilience that is necessary to face the current adversities	Attempt to define shared goals for the therapeutic process create an initial diagnostic framework identify unprocessed or unregulated emotions	Psychoeducation on the meaning of emotions, their manifestation in the body (e.g., heartbeat, breath, muscles, bowels) and on the functioning of the worried mind (e.g., anxious and catastrophic thoughts)
3	Practice emotion regulation consolidate the sense of self-efficacy and self-control identify resources and vulnerabilities	Validate the intrapsychic and interpersonal resources associated with a greater degree of adaptation to the stressful situation, including: a flexible personality; positive beliefs about the self; identity roles and acceptance and commitment skills; work functioning; solid network of friends; family/loved ones	Bringing the mind back to the “*here and now*” versus the past and the future, and other mindfulness concepts sensory-motor techniques (e.g., tolerance window, grounding, posture modification, breathing) stabilization techniques, for example “safe place” body scanning and guided relaxation techniques enhancement of resilience
4–6	Address areas of clinical concern investigate defense mechanisms	*Traumatic and post-traumatic symptoms* validation, regulation techniques and resource consolidation exploration of sensations, emotions, movements, thoughts (such as hyperarousal, intrusiveness/surrendering, avoidance, hyper-compensation)	Imagery rescripting with a support figure that is able to mitigate the guilt/shame preponderance security, protection and care for one’s own needs cognitive restructuring on “beliefs,” cognitive biases, compassionate self-representation recognition of improvements that were made by the patients with their own resources.
		*Grief* navigate the phases of emotional processing: narration of the event; grounding, emotional regulation; modulation of motivational and affective systems; evolution of defense mechanisms from the most primitive (dissociation, denial) to the most advanced (depression)	Vocalization of suffering and emotional expressiveness awareness on defense mechanisms that tend to repress the memory processing of abandonment feelings and blaming tendencies representation remodeling with the respect to the relationship with the lost ones, with the goal of mitigating suffering
		*Triggered or exacerbated psychiatric symptoms* contextualizing the occurrence of known or new psychiatric symptoms assigning meaning to the worsening of such symptoms	Retracing the patient’s history mentalization mood modulation and emotional self-regulation
7	Integrate the lived experience in the cohesive narrative of the self	Recognize patient’s emotions/behaviors experienced during the acute phase as their own.	Validation of mental states and thought patterns experienced during the acute phase experience reformulation recognizing dualism whenever rethinking about the lived experiences – promoting dialectical thinking acceptance of new limitations and life adaptations
8	Discuss internal working models or relational patterns that have emerged during therapy closure	Summary of the therapeutic strategies that have been discussed during the sessions	Psychoeducation on relapse prevention description of risk mitigation strategies

#### Session 1

This session includes introductions, exploration of the patient’s current experience space, and identification of the areas of suffering on the basis of the COVID-19 event. The narrative of the COVID-19 experience covers events of contagion, hospitalization, remission, recovery for both patients and family members. Information is collected regarding health conditions, clinical severity, length, and type of hospitalization, as well as particularly difficult moments. Space is dedicated to the float back of stressful events related to COVID-19 (situations, places, people, and images). The therapist is invested in forming the bases for therapeutic alliance, and the session usually ends with the validation of the suffering, an explicit offering of closeness and support on the part of the therapist, and an attempt to define shared goals for the therapeutic process.

#### Session 2

The information collected in the first session allows the therapist to create an initial diagnostic framework – both at a structural and functional level – and to identify unprocessed or unregulated emotions. In the context of the second interview, the therapist attempts to confirm the hypothesis developed during the first meeting and makes explicit their clinical goal. We believe this step is fundamental given the short duration of the intervention, so that patient and therapist can focus and work together more effectively. The shared goal is centered around learning to regulate disruptive emotions, in order to reach the level of resilience that is necessary to face the current adversities. Concomitantly, the therapist offers psychoeducation on the meaning of emotions, their manifestation in the body (heartbeat, breath, muscles, and bowels) and on the functioning of the worried mind (anxious and catastrophic thoughts).

#### Session 3

During session three, the therapist usually presents and practices techniques for emotion regulation, including: (i) bringing the mind back to the here and now versus the past and the future, and other mindfulness concepts; (ii) sensory motor techniques (e.g., tolerance window, grounding, posture modification, and breathing); (iii) stabilization techniques, for example “safe place”; (iv) body scanning and guided relaxation techniques; and (v) enhancement of resilience. In the event that patients have more resources or have demonstrated that they have self-monitoring skills and/or that they have successfully differentiated emotions at play, these emotion regulation techniques can be presented during session 2. Finally, with the aim of consolidating the sense of self-efficacy and self control that often derives from it, the therapist identifies and validates the intrapsychic and interpersonal resources associated with a greater degree of adaptation to the situation, including: a flexible personality; positive beliefs about self; identity roles and acceptance and commitment skills; work functioning; solid network of friends; family/loved ones.

#### Sessions 4–6

Based on the clinical material that has emerged in the first three sessions, the diagnostic framework and the identification of resources and vulnerabilities, the clinical macro-areas are addressed while respecting the patient’s way of being as well as defense mechanisms.

##### Traumatic and Post Traumatic Symptoms

Certainly, the psychological picture is strictly dependent on the clinical conditions of the patient himself or of the family member:

– If the clinical conditions are serious, fluctuating, or worsening (active trauma), we believe it is appropriate to continue with validation, regulation techniques and resource consolidation as regulating emotions remains the most valuable tool in such circumstances. If new emotion regulation techniques are experienced and used successfully, they can become new valuable tools in the hands of the patient. It is useful to identify critical moments (e.g., the moment before going to bed and falling asleep; before and after the phone call from the hospital for family members), and at the same time imagine how to use emotional resources in such moments.
– If the clinical conditions are improving, there is more space to take distance from the emergency and observe it from afar (post-trauma). In general, we propose a work where used resources are recognized, and it becomes possible to face particularly challenging moments without reliving them (maintaining the “double focus”). By now, the exploration of sensations, emotions, movements, thoughts (such as hyperarousal, intrusiveness/surrendering, avoidance, hyper compensation) is usually more fluid, and allows the treatment and re-processing of the traumatic experience. The following techniques can be used: (i) Imagery rescripting with a support figure that is able to mitigate the guilt/shame preponderance; (ii) security, protection and care for one’s own needs; (iii) cognitive restructuring on “beliefs,” cognitive biases, compassionate self-representation; and (iv) recognition of improvements that were made by the patients with their own resources.

##### Grief

The experience of grief is treated incrementally, maintaining an attitude of empathic support and accompaniment during the various phases of emotional processing. These phases include: narration of the event; grounding, emotional regulation; modulation of motivational and affective systems; evolution of defense mechanisms from the most primitive (dissociation, denial) to the most advanced (depression). Given the brief duration of the interventions, realistic goals that can be set include the development of tolerance for suffering and the ability to attribute meaning to the event.

##### Psychiatric Symptoms Triggered and/or Exacerbated by the COVID-19 Pandemic

In our experience, the analysis of the emotions triggered by COVID-related events brings to the surface internal working models that have already emerged in the history of patients, such as the sense of guilt or the feeling of frustration for not being able to be useful. This analysis seems to facilitate the connection between the current lived experience and other difficult moments of life, thus allowing the experience to be “absorbed” and perceived as coherent to the self. Psychiatric patients who develop COVID-19 symptoms usually experience an exacerbation of pre-existing symptomatologic traits. We believe that retracing the patient’s history, contextualizing the occurrence of known or new psychiatric symptoms, and ultimately assigning meaning to the worsening of such symptoms are useful management techniques that can help patients navigate the challenge and develop stronger skills.

#### Sessions 7–8

The last two sessions are dedicated to the process of integrating the lived experience in the cohesive narrative of the self. Unless there are specific open topics or needs, patients are encouraged to recognize the emotions/behaviors they had during the acute phase as their own. Sometimes this process already begins in previous sessions, and some patients recognize their own internal working models when narrating COVID-19 related moments that they have recently experienced. For example, when her spouse was discharged from the ICU and returned home, a patient said that she was surprised by how bothered she felt toward the newly-found and much-desired closeness with her husband. During the sessions, she developed the ability to recognize the feeling of seeing her personal space invaded as a coping mechanism that she had always put in place after long periods of distance from loved ones (after the holidays away from her daughter or after her husband’s business trip).

The last session (Session 8) usually includes psychoeducation on relapse prevention and alert signs, especially around the symptoms of anxiety (panic attacks, somatization) and depression (rumination, mood). Along with risk mitigation strategies, patients are offered a summary of the therapeutic strategies that have been discussed during the sessions, and encouraged to reflect on the tools they can now use if and when symptoms arise in the coming future. Undeniably, relapse prevention and closure of the therapeutic process poses some critical challenges: in the face of a symptomatic improvement, a brief psychotherapy program can reveal internal working models or relational patterns worthy of further understanding and treatment, especially for those with dysfunctional personality traits and low emotional competency. However, we believe that the eschatological power of this brief therapeutic program centered on COVID-19 related experiences precisely lies in the possibility of generating in patients a desire for growth and awareness that goes beyond the adversities of the present moment.

## Final Considerations

The opportunities presented by technology to disseminate new psychological models have revolutionized the field of mental health. Tele-psychiatry services make it possible to overcome the barriers posed by traditional mental health services during and after hospitalization of COVID-19 patients. Different lines of evidence indicate that different age groups, regardless of educational levels and psychopathological characteristics, have enough tech literacy to handle video calls and engage successfully with therapists. Using the support of technology to deliver psychotherapeutic services has been largely experimented in high-income countries during the COVID-19 pandemic. Despite obvious advantages in the continuity of treatments during restrictions, tele-psychiatry services face some limitations. As Liem et al. (2020) highlighted in a recent article, confidentiality, competency, compliance, consent and contingency should be addressed as ethical standards for tele-mental health. Once these aspects are addressed and the technological infrastructure is in place, the response to the COVID-19 emergency is likely to go beyond a temporary increase in the use of tele-psychiatry, and could represent the basis for a future standard of psychological assistance in the course of emergency situations that require restriction measures in domestic and social welfare contexts.

A plethora of services, interventions, tools has been redirected and used in response to COVID-19, most of which fell outside our database and literature search. Albeit narrow in scope, our endeavor was to exclusively find theory-driven models and protocols that were studied in a research environment. Namely, we were interested in developing, describing and illustrating here an intervention model that is based on the principles of psychotherapeutic practice, scientifically sound and easily scalable. We believe that non-specific psychological programs, tools, or interventions often do not meet the standards of psychotherapeutic practice, thus limiting the depth and breadth of the clinical work that the psychopathology of COVID-19 patients often requires.

Because the proposed intervention results from the integration of components from a variety of therapeutic approaches, and because it is designed to be disseminated on a large scale, we have attempted to identify characteristics and qualifications that therapists should have in order to deliver this treatment. In recent years, a large number of studies have tried to identify the therapist-related characteristics that are significantly associated with psychotherapy outcome factors (Mulder et al., 2017). Some of them seem to be common across different psychotherapeutic theoretical backgrounds and methodologies, since they are more related to therapy goals such as motivation to change. Nevertheless, some therapist characteristics are indispensable for the delivery of this intervention, including: (i) motivation and experiences with care with older patients and patients with severe medical comorbidities, (ii) expertise in collaborating with medical professionals (ICU doctors, pneumologists, etc…), and (iii) competency in treating different psychological manifestations in acute or post-acute phases. Moreover, our experience suggests that therapists might face resistance from COVID-19 patients because psychological treatment is not always actively requested by the patients themselves. Given the heterogeneity of psychological and medical complexity among COVID-19 survivors and family members and the customized approach we have developed, we believe that psychiatrists and clinical psychologists are the most suited mental health professionals to be trained on this treatment model.

The goal of this manuscript was to present the development process, the target population, as well as the manualized content of our psychotherapeutic program. This initiative is part of a larger project that will sequentially evaluate acceptability, feasibility, efficacy and effectiveness. A pilot study is currently underway to evaluate the feasibility and preliminary efficacy of this treatment. Interim results seem to indicate good acceptability, with virtually absent attrition rates and high attendance rates from COVID-19 patients and their family members. Clinicians involved in the project found the delivery method highly acceptable and compatible with their work flow. To date, no adverse events have been reported.

We believe that the intervention model described here has great innovation potential in that: (1) it offers immediate psychotherapeutic support to all those who live experiences of psychological suffering associated with COVID-19; (2) helps therapists to operate in acute and subacute settings, overcoming the barriers imposed by public health and prevention measures; and (3) contributes to study and determine the procedures by which tele-psychotherapy can be best implemented.

## Methodological Significance

This manuscript leverages ongoing clinical experiences with COVID-19 survivors and family members to propose a structured 8-week brief psychotherapy program designed to address commonly endorsed mental health symptoms associated with COVID-19.

## Data Availability Statement

The original contributions presented in the study are included in the article/supplementary material, further inquiries can be directed to the corresponding author/s.

## Author Contributions

BB, SZ, CF, and VG: conceptualization, investigation, methodology, and writing – review and editing. MB and CB: writing – original draft, investigation, methodology, and writing – review and editing. PB: conceptualization, methodology, and writing – review and editing. All authors contributed to the article and approved the submitted version.

## Conflict of Interest

BB serves as a Consultant for Posit Science, a company that produces cognitive training and assessment software. The remaining authors declare that the research was conducted in the absence of any commercial or financial relationships that could be construed as a potential conflict of interest.

## Publisher’s Note

All claims expressed in this article are solely those of the authors and do not necessarily represent those of their affiliated organizations, or those of the publisher, the editors and the reviewers. Any product that may be evaluated in this article, or claim that may be made by its manufacturer, is not guaranteed or endorsed by the publisher.

## Footnotes

1
https://ourworldindata.org/coronavirus-data?country=∼ITA

2
https://opendatadpc.maps.arcgis.com

3
https://innovazione.gov.it/notizie/articoli/en/tele-medicine-and-monitoring-systems-a-call-for-technologies-to-contrast-the-spre/
